# Self‐defined former smokers consume the highest opioid quantities following knee and shoulder arthroscopy

**DOI:** 10.1002/ksa.12403

**Published:** 2024-08-06

**Authors:** Hassaan Abdel Khalik, Ajaykumar Shanmugaraj, Seper Ekhtiari, Nolan S. Horner, Aaron Gazendam, Nicole Simunovic, Olufemi R. Ayeni, NO PAin Investigators

**Affiliations:** ^1^ Department of Surgery, Division of Orthopaedics McMaster University Hamilton Ontario Canada; ^2^ University of British Columbia Faculty of Medicine Vancouver British Columbia Canada; ^3^ Department of Surgery, Division of Orthopaedic Surgery University of Toronto Toronto Ontario Canada; ^4^ Genesis Orthopedics and Sports Medicine Chicago Illinois USA

**Keywords:** knee arthroscopy, multimodal analgesia, opioid consumption, postoperative pain, shoulder arthroscopy

## Abstract

**Purpose:**

To identify risk factors associated with increased postoperative opioid consumption and inferior pain outcomes following knee and shoulder arthroscopy.

**Methods:**

Using the data set from the NonOpioid Prescriptions after Arthroscopic Surgery in Canada (NO PAin) trial, eight prognostic factors were chosen a priori to evaluate their effect on opioid consumption and patient‐reported pain following arthroscopic knee and shoulder surgery. The primary outcome was the number of oral morphine equivalents (OMEs) consumed at 2 and 6 weeks postoperatively. The secondary outcome was patient‐reported postoperative pain using the Visual Analogue Scale (VAS) at 2 and 6 weeks postoperatively. A multivariable linear regression was used to analyse these outcomes with eight prognostic factors as independent variables.

**Results:**

Tobacco usage was significantly associated with higher opioid usage at 2 (*p* < 0.001) and 6 weeks (*p* = 0.02) postoperatively. Former tobacco users had a higher 2‐week (*p* = 0.002) and cumulative OME (*p* = 0.002) consumption compared to current and nonsmokers. Patients with a higher number of comorbidities (*p* = 0.006) and those who were employed (*p* = 0.006) reported higher pain scores at 6 weeks. Patients in the ‘not employed/other’ category had significantly lower pain scores at 6 weeks postoperatively (*p* = 0.046).

**Conclusion:**

Former smoking status was significantly associated with increased post‐operative opioid consumption following knee and shoulder arthroscopy at 2 and 6 weeks postoperatively. Increased pain was found to be significantly associated with employment status and an increasing number of comorbidities at 6 weeks postoperatively. These findings can aid clinicians in identifying and mitigating increased opioid utilization as well as worse pain outcomes in high‐risk patient populations.

**Level of Evidence:**

Level III, cohort study.

**Abbreviations**
ANOVA: analyses of variance
BMI: body mass index
NSAID: nonsteroidal anti‐inflammatory drug
OME: oral morphine equivalent
OR: odds ratio
RCT: randomized controlled trial
SD: standard deviation
VAS: Visual Analogue Scale

## INTRODUCTION

The on‐going opioid epidemic, both in the United States and globally, has resulted in a growing body of research, including several randomized controlled trials (RCTs), exploring various opioid reduction strategies following orthopaedic surgery including preoperative education, adjunct analgesia and multimodal analgesia have found promising results [9, 11, 22, 30, 32]. Though encouraging, not all patients may be equally amenable to standardized nonopioid analgesic strategies which highlights the need for additional patient‐specific protocols.

While preoperative opioid use has been established as a strong predictor for postoperative opioid use, limited high‐quality literature exists identifying additional risk factors for acute postoperative pain and opioid use following knee and shoulder arthroscopy. Retrospective reviews have identified a combination of preoperative opioid use, posttraumatic stress disorder, smoking, chronic pulmonary obstructive disorder and more severe grades of osteoarthritis as risk factors for chronic opioid utilization following knee arthroscopy [12, 27]. Still, there remains a paucity of prospectively collected evidence as it pertains to risk factors predictive of postoperative pain and on‐going opioid use in patients undergoing knee and shoulder arthroscopy.

The NonOpioid Prescriptions after Arthroscopic Surgery in Canada (NO PAin) trial is a multicentre RCT that demonstrated, in comparison to standard of care, a nonopioid multimodal analgesic protocol significantly reduced opioid consumption while maintaining appropriate pain levels in patients that underwent knee and shoulder arthroscopy [22]. Nonetheless, the NO PAin trial did not identify risk factors for increased opioid utilization or inferior pain outcomes that can guide clinicians to leverage the aforementioned opioid‐sparing protocol in a targeted, resource‐efficient manner. Therefore, the purpose of the current study is to utilize this trial's prospectively collected data to identify risk factors associated with increased pain and opioid use in the acute 6‐week postoperative period. It was hypothesized that male sex, age younger than 50, on‐going regular alcohol use or smoking, increased number of comorbidities and operative time greater than 90 min would be associated with increased postoperative pain and opioid use.

## MATERIALS AND METHODS

### NO PAin trial design

The NO PAin trial was registered at Clintrials.gov (NCT04566250). Ethics approval for this study was granted by the Hamilton Integrated Research Ethics Board (Version 1.0, 14‐January‐2021, HIREB #12–670). The methodology of this trial has been published previously [6]. The trial was performed across three sites in Hamilton, Ontario: McMaster University Medical Centre (MUMC), St. Joseph's Healthcare and the Hamilton General Hospital to evaluate the efficacy of a nonopioid pain management modality for patients aged 18 years and older undergoing outpatient knee or shoulder arthroscopy. Patients were excluded if they met any of the following exclusion criteria: (1) preoperative chronic opioid use; (2) undergoing surgery for a workers' compensation claim or having any other active litigation for a workplace injury; (3) operative time greater than 3 h; (4) requiring postoperative overnight admission to hospital; and/or (5) contraindication or allergy to acetaminophen, nonsteroidal anti‐inflammatory drugs, hydromorphone or morphine.

The previously published NO PAin trial consisted of 200 patients that were randomized to the control (standard of care) or intervention (nonopioid prescription and infographic) group [22]. The intervention group consisted of patients receiving a standardized protocol comprised of the following: (1) 500 mg of naproxen taken orally twice a day as needed (number of tablets: 60; dose per tablet: 500 mg tablets) and 40 mg of pantoprazole taken orally while consuming naproxen; (2) 1000 mg of acetaminophen taken orally every 6 h as needed (number of tablets: 100; dose per tablet: 500‐mg tablets); (3) opioid rescue prescription consisting of 1 mg of hydromorphone taken orally every 4 h as needed (number of tablets: 10; dose per tablet: 1‐mg tablets); and (4) patient educational infographic with further information of both pharmacologic and nonpharmacological pain management strategies. In regards to the rescue opioid, patients were instructed to take this form of analgesia only when the nonopioid protocol was ineffective in providing adequate pain relief.

The primary outcome was the number of oral morphine equivalents (OMEs) consumed at 6 weeks postoperatively, quantified using a patient‐reported medication diary. OMEs allow for the standardization of consumed opioids in milligrams across various opiates. Secondary outcomes included (1) patient‐reported pain using a Visual Analogue Scale (VAS), (2) patient‐reported satisfaction with pain control using one question from the Hospital Consumer Assessment of Healthcare Providers and Systems questionnaire, (3) number of OMEs prescribed, (4) number of opioid refills and (5) any adverse events up to 6 weeks postoperatively (i.e. gastrointestinal upset, drowsiness, dizziness, etc.).

### Selection of prognostic factors

Based on biologic rationale and previous reports in the literature, the authors identified potential prognostic factors from the baseline demographics and surgical data collected as part of the NO PAin trial [22]. Predictors with fewer than 10 events (or 10 observations for each predictor variable in linear regression) were not chosen to prevent overfitting, unstable models [23]. The minimum number of patients that completed follow‐up at each time point for each outcome was 178. Therefore, an observation rate of 178 was used and a total of eight prognostic factors (12 levels) were selected and included age (continuous), sex (male, female), body mass index (BMI) (continuous), tobacco use (none, current smoker, self‐defined former smoker), alcohol use (none, <5 drinks per week, ≥5 drinks per week), number of comorbidities (continuous), employment status and operative time (continuous variable). Tobacco use status was self‐defined as (1) none, (2) current smoker and (3) self‐defined former smoker; no formal criteria were provided to patients for self‐categorization. Employment status was categorized as (1) employed, (2) students, (3) retired or (4) unemployed/other which includes homemakers, patients off work based on doctor's advice, as well as unemployed patients.

### Statistical analysis

The primary analysis for this study was a multivariable linear regression using the number of OMEs consumed at 2 and 6 weeks postoperatively as the dependant variable. All tests were two‐tailed with *⍺* = .05. Secondary analyses included a multivariable linear regression with VAS pain scores at 2 and 6 weeks postoperatively as the dependant variable. Analyses of variance were conducted on categorical dependant variables found to be significant predictors of the model to assess whether statistically significant differences existed across the said dependant variables' categories. Descriptive data for continuous variables were summarized using means and standard deviations (SDs), while categorical variables were summarized with counts and percentages. All analyses were performed by a senior clinician with a statistical background (SE) using SAS version 9.4.

## RESULTS

### Participant characteristics

There were 694 patients undergoing knee or shoulder arthroscopy with 494 excluded due either not meeting the exclusion criteria (*n* = 214), declining to participate (*n* = 49) or for other reasons (*n* = 231) such as being unable to contact the patient, being enroled in a competing trial or surgery being delayed outside of the study window [22]. Of the 200 patients included in the NO PAin trial, seven patients cancelled their surgery after randomization and were thus excluded from the final analysis, for a total of 95 patients in the opioid‐sparing group and 98 in the standard of care group. Included patients were primarily male (62.5%), with a mean age of 43.0 (SD, 15.3) years and a mean BMI of 29.2. The majority of patients underwent knee surgery (*n* = 143; 73.1%). The most common procedures performed were a meniscectomy (*n* = 93; 48.2%) in the knee and biceps tenotomy (*n* = 25; 13.0%) in the shoulder. Mean operative time in minutes was 48.6 min (SD, 32.0) and was similar between the two groups [22].

### Primary outcome

#### Factors associated with number of OMEs consumed

A total of 180 and 172 patients had reported OMEs consumed at the 2 and 6 weeks follow‐up, respectively. Tobacco use was significantly associated with number of OMEs consumed at 2 (*p* < 0.001) and 6 weeks (*p* = 0.02) postoperatively (Table 1). Former tobacco users showed greater OME consumption at 2 (*p* = 0.002) and 6 weeks postoperatively (*p* = 0.002) compared to current and nonsmokers (Table 2). The remaining prognostic factors were not significantly associated with number of OMEs consumed at 2 and 6 weeks postoperatively.

**Table 1:** Opioid consumption multivariable linear regression analysis outcomes.

	2 weeks postoperatively		6 weeks postoperatively	
Variable	*β* Coefficient (95% CI)	*p* Value	*β* Coefficient (95% CI)	*p* Value
Patient demographics				
Age	−0.102	0.220	−0.098	0.248
Sex	0.105	0.178	0.097	0.225
BMI	−0.111	0.156	−0.100	0.215
Tobacco use	0.250	<0.001	0.239	0.002
Alcohol use	−0.002	0.980	0.010	0.902
Number of comorbidities	0.082	0.290	0.093	0.239
Employment status	−0.071	0.366	−0.067	0.408
Surgical factors				
Operative time	0.094	0.264	0.097	0.262

Abbreviations: BMI, body mass index; CI, confidence interval.

**Table 2:** Tobacco use status and opioid consumption.

	OME at 2 weeks		OME at 6 weeks	
Tobacco use status	Number of patients	Mean ± SD	Number of patients	Mean ± SD
None	151	35.9 ± 65.1	145	36.8 ± 66.3
Current smoker	18	31.1 ± 49.7	16	24.8 ± 39.3
Former smoker	12	111.2 ± 143.0	12	111.2 ± 143.0
Total	181	40.4 ± 73.3	173	40.86 ± 74.2
ANOVA *p* value		0.002^a^		0.002^a^

Abbreviations: ANOVA, analysis of variance; OME, opioid milliequivalents; SD, standard deviation.

^a^ Statistically significant.

### Secondary outcomes

#### Factors associated with VAS pain scores

A total of 183 and 176 patients had reported VAS scores at the 2‐ and 6‐week follow‐up, respectively. Number of comorbidities (*p* = 0.006) and employment status (*p* = 0.006) were significantly associated with higher VAS scores (i.e., higher pain) at 6 weeks (Table 3). At 6 weeks postoperatively, employed patients reported the highest VAS pain scores while patients in the ‘not employed/other’ category presented with the lowest VAS scores; students and retired patients reported VAS scores in between the two aforementioned categories (*p* = 0.046) (Table 4).

**Table 3:** VAS multivariable linear regression analysis outcomes.

	2 weeks postoperatively		6 weeks postoperatively	
Variable	*β* Coefficient (95% CI)	*p* Value	*β* Coefficient (95% CI)	*p* Value
Patient demographics				
Age	−0.066	0.441	−0.007	0.937
Sex	0.002	0.982	0.106	0.173
BMI	−0.093	0.246	−0.089	0.258
Tobacco use	−0.028	0.711	0.039	0.602
Alcohol use	−0.071	0.373	0.076	0.327
Number of comorbidities	0.103	0.198	0.212	0.006
Employment status	−0.071	0.374	−0.218	0.006
Surgical factors				
Operative time	−0.062	0.473	−0.111	0.189

Abbreviations: BMI, body mass index; CI, confidence interval; VAS, Visual Analogue Score.

**Table 4:** Employment status and VAS score.

	VAS at 2 weeks		VAS at 6 weeks	
Employment status	Number of patients	Mean ± SD	Number of patients	VAS at 6 weeks, mean ± SD
Employed	131	17.2 ± 19.8	125	15.8 ± 19.8
Retired	21	16.6 ± 15.1	21	11.3 ± 12.3
Student	20	16.0 ± 24.1	20	7.7 ± 12.2
Not employed, other	12	12.7 ± 21.0	11	3.3 ± 3.7
Total	184	16.7 ± 19.8	177	13.6 ± 18.0
ANOVA *p* value		0.899		0.046^a^

Abbreviations: ANOVA, analysis of variance; SD, standard deviation; VAS, visual analogue scale.

^a^ Statistically significant.

## DISCUSSION

The primary finding of this study was that tobacco use status was significantly correlated with increased opioid consumption at 2 and 6 weeks postoperatively. More specifically, patients with a prior history of tobacco use had higher postoperative opioid consumption relative to nonsmokers and current smokers. Correlation analysis of the VAS pain score demonstrated that an increased number of comorbidities and employment status were the only two predictive factors for increased pain at 6 weeks postoperatively. Specifically, being employed resulted in the highest VAS pain scores and being unemployed resulted in the lowest scores.

The evidence is conflicting regarding the association between smoking status and postoperative opioid consumption following arthroscopic procedures. While this study's analysis demonstrated a statistically significant association between smoking status and opioid consumption, a prior cohort study by Scarcella et al. in patients undergoing knee arthroscopy demonstrated no such correlation [28]. Despite this difference, Scarcella et al.'s study found a similar trend of former smokers having a higher odds of increased opioid consumption compared to current smokers (odds ratio [OR] 1.93, 95% confidence interval [CI]: 0.53–7.56) with no difference between nonsmokers and current smokers (OR: 1.05, 95% CI: 0.31–3.78). Similar to this study, no formal temporal threshold was used to define former smokers. When considering that the analgesic effects of nicotine are mediated by both nicotinic and μ‐opioid receptors, it is possible that patients who recently quit smoking may be at increased risk of postoperative opioid use to compensate for the lack of nicotine intake and its analgesic benefits [5]. Conversely, another database study by Ridenour et al. evaluating patients undergoing knee arthroscopy demonstrated that tobacco use is associated with increased postoperative opioid consumption up to 12 months postoperatively, though their study did not analyse the effect of being a former smoker [26]. Orthopaedic surgeons will be able to identify tobacco users and advise on smoking cessation due to the known inferior postoperative outcomes following arthroscopic surgery [10, 18]. This analysis suggests that former smokers may be identified with the goal of providing them with additional analgesic strategies to minimize postoperative opioid consumption.

A statistically significant correlation was found between number of comorbidities and inferior VAS pain outcomes at 6 weeks postoperatively. As per the index trial, back pain, asthma and depression were the three most common comorbidities in the included patient population [22]. While further subgroup analysis was not possible due to this study's sample size, the impact of comorbidities has been thoroughly studied in the arthroscopic patient population. Following shoulder arthroscopy, an increased number of comorbidities has been demonstrated to be associated with worse pain and patient‐reported outcomes following rotator cuff repair [24], as well as less favourable outcomes following manipulation under anaesthesia for frozen shoulder [16]. Another important relationship highlighted by Rhon et al. is the development of comorbidities following arthroscopic surgery, which may contribute to less favourable long‐term outcomes. Their database study found increased age and lower socioeconomic status was associated with a higher relative risk of developing postoperative comorbidities [25]. Ultimately, while arthroscopic procedures improve a patient's quality of life, patients with higher preoperative comorbidities are at increased risk of inferior outcomes. Rigorous screening and the optimization of preoperative comorbidities using an interdisciplinary approach may be effective in reducing their adverse impact, though formal research assessing such strategies from both clinical and cost‐effective perspectives is warranted.

This study also demonstrated that employment status is a statistically significant predictor of increased VAS pain scores at 6 weeks postoperatively. Specifically, employed patients present with higher pain scores relative to students, retirees and unemployed patients. A study by Nadarajah et al. across 319 patients undergoing various shoulder procedures, most of which were arthroscopic, demonstrated that employed patients had the highest pretreatment expectations [20]. While prior literature has demonstrated that higher preoperative expectations are associated with higher patient satisfaction scores, these studies are usually long term in nature [21]. As such, a discordance between patient expectations and outcomes in the acute postoperative period may be responsible for this study's findings. Another explanation is that employed patients may be returning to work relatively soon following surgery and possibly exacerbating their postoperative pain. More specifically, patients may be consistently taking the first two postoperatively weeks off from work and returning thereafter resulting in increased pain at 6 weeks postoperatively. Ultimately, this phenomenon is multifactorial and future studies on the subject matter may benefit from collecting more granular data on return‐to‐work timing as well as additional factors that may drive an earlier return‐to‐work timing (i.e. sole breadwinner, salaried versus hourly wages, etc.). It is important to note that while the differences in VAS scores were statistically significant, they did not surpass prior reported minimally important clinical differences in orthopaedic surgery [3, 14, 19]. Furthermore, the generalizability of these findings is limited to evaluation during the early postoperative period, and future exploratory analysis may be warranted to further assess this trend.

From a clinical standpoint, this study identifies patient populations at higher risk for increased opioid utilization and inferior pain outcomes in the acute postoperative period that may benefit from opioid‐sparing analgesic protocols. As demonstrated by the NO‐PAin trial, a multimodal analgesic regimen is effective in reducing postoperative opioid consumption following both knee and shoulder arthroscopy with no statistically significant differences in patient pain or satisfaction scores [22]. Preoperative patient education is another effective modality to decrease postoperative opioid use following arthroscopic surgery with high‐quality evidence confirming its efficacy [2, 30]. Such tools can be coupled with novel prognostic methodologies to identify high‐risk patient populations warranting adjunct analgesic strategies. For example, a recent retrospective study by Ueki et al. demonstrated that a decreased pain threshold is associated with inferior functional outcomes following arthroscopic rotator cuff repair and that even weak opioids can effectively improve patients' pain thresholds in the acute postoperative period [31]. Additionally, machine‐learning algorithms continue to be developed to aid clinicians more accurately identify high‐risk patients [15]. Ultimately, several tools exist for minimizing opioid consumption following arthroscopic surgery and future research is warranted to assess how to best amalgamate these strategies for optimal outcomes in a resource‐efficient manner.

### Limitations

This study is not without its limitations. Although robust in size, the inclusion of 193 patients prevents a more granular analysis across various predictive factors. Nonetheless, larger database studies can be used to augment this study's findings and inform future‐focused trials [26]. Second, the short follow‐up period prevents further assessment of prolonged postoperative opioid use beyond 6 weeks postoperatively. This appears to be a common limitation of prospectively collected evidence on the subject matter and highlights the need for future long‐term studies [4, 11, 17]. Comparison to long‐term studies is rendered difficult due to several core methodological differences including the utilization of registry databases as well as often including patients with preoperative opioid in the analysis, a major confounding variable [1, 4, 7]. Third, this study excluded patients with a prior history of opioid utilization, a known predictive factor for increased postoperative opioid consumption [26, 29]. While this allowed for a focused analysis on the opioid‐naïve patient, this study's findings are not generalizable to patients with a history of chronic opioid use. Fourth, opioid consumption data was based on patient‐reported diaries, which are susceptible to error, though this methodology has been consistently utilized in various opioid‐related orthopaedic literature [8, 13]. Finally, surgeries associated with a workers' compensation claim were excluded from the primary study thus precluding inclusion in this analysis. Due to this patient population having increased postoperative opioid utilization, it is not uncommon for it to be excluded from similar prognostic studies [33]. Despite these limitations, this analysis builds upon the NO PAin trial's prospectively collected data to shed light on patient risk factors associated with increased opioid use and increased pain postoperatively.

## CONCLUSION

Former smoking status was significantly associated with increased postoperative opioid consumption following knee and shoulder arthroscopy at 2 and 6 weeks postoperatively. Increased pain was found to be significantly associated with employment status and an increasing number of comorbidities at 6 weeks postoperatively. The findings of this analysis can further aid clinicians in identifying and mitigating increased opioid utilization as well as worse pain outcomes in high‐risk patient populations undergoing arthroscopic surgery.

## AUTHOR CONTRIBUTIONS

**Hassaan Abdel Khalik**: Data preparation; manuscript preparation; manuscript editing. **Ajaykumar Shanmugaraj**: Manuscript preparation; manuscript editing. **Seper Ekhtiar**i: Statistical analysis; manuscript editing; content expert. **Nolan S. Horner**: Manuscript editing; content expert. **Aaron Gazendam**: Manuscript editing; content expert. **Nicole Simunovi**: Statistical analysis; manuscript editing. **Olufemi R. Ayeni**: Supervision of project; idea for project; manuscript editing; content expert. All authors were involved in drafting and proofreading the final manuscript.

## NO PAin Investigators

Writing Committee: Hassaan Abel Khalik, MD, MMI (McMaster University), Ajaykumar Shanmugaraj, MD(c), BHSc (University of British Columbia), Seper Ekhtiari, MD, MSc (McMaster University, University of Toronto), Nolan S. Horner, MD, FRCSC (Genesis Orthopedics and Sports Medicine), Aaron Gazendam, MD, MSc, FRCSC (University of Toronto), Nicole Simunovic, MSc (McMaster University), Olufemi R. Ayeni, MD, PhD, FRCSC (McMaster University). Methods Centre (McMaster University): Olufemi R. Ayeni, MD, PhD, FRCSC (Co‐Principal Investigator); Seper Ekhtiari, MD, MSc (Co‐Principal Investigator, Statistician), Aaron Gazendam, MD (Co‐Principal Investigator), Nolan S. Horner, MD, FRCSC (Co‐Principal Investigator), Nicole Simunovic, MSc (Research Manager); Andrew Duong, MSc, Andrea K. Ponniah, BSc (Project Management). Steering Committee: Olufemi R. Ayeni, MD, PhD, FRCSC (Chair, McMaster University), Nolan S. Horner, MD, FRCSC (McMaster University), Aaron Gazendam, MD (McMaster University), Seper Ekhtiari, MD, MSc (McMaster University), Caitlin VanDeCapelle, MD, FRCPC (Hamilton Health Sciences), Franca Mossuto, RN (Hamilton Health Sciences), Eric Romeril, RPh, ACPR, BScPharm (Hamilton Health Sciences), Steve Phillips, MSc (Fergus, ON‐Patient Advisor). Adjudication: Herman Johal, MD, MPH, FRCSC (Chair); Jamal Al‐Asiri, MD, FRCSC; Daniel Tushinski, MD, FRCSC (McMaster University). Medical Monitor: Thomas J. Wood, MD, MSc FRCSC (McMaster University). Participating Clinical Sites. Canada: McMaster University Medical Centre (Hamilton, ON)—Olufemi R. Ayeni MD, PhD, FRCSC; Darren L. de SA MBA(c), MD, FRCSC; Seper Ekhtiari, MD, MSc; Aaron Gazendam, MD; Nolan S. Horner, MD, FRCSC; Devin Peterson, MD, FRCSC; Andrew Duong, MSc; Andrea K. Ponniah, BSc; Nicole Simunovic, MSc. Hamilton General Hospital (Hamilton, ON)—Matthew Denkers, MD, FRCSC; Andrew Duong, MSc; Andrea K. Ponniah, BScSt. Joseph's Healthcare Hamilton (Hamilton, ON)—Anthony Adili, MD, FRCSC; Moin Khan, MD, MSc, FRCSC; Vickas Khanna, MD, FRCSC, MHA; Jaydeep Moro, MD, FRCSC; Kim Madden, PhD; Imad Kashir BSc; Grace Mwakijele, BPH; Darren Young Shing, BHSc.

## CONFLICTS OF INTEREST STATEMENT

O. R. A. is on the speaker bureau for Stryker Canada and is the editor‐in‐chief of JISAKOS, Tier 2 Canada Research Chair in Joint Preservation and president/owner of Notch Academy. The remaining authors declare no conflict of interest.

## ETHICS STATEMENT

Ethical approval for this study was granted by the Hamilton Integrated Research Ethics Board (#12670). Written informed consent was obtained from all participants.

## Data Availability

The data that support the findings of this study are not openly available and are available from the corresponding author upon reasonable request.
